# Evidence for anisotropic spin-triplet Andreev reflection at the 2D van der Waals ferromagnet/superconductor interface

**DOI:** 10.1038/s41467-021-27041-w

**Published:** 2021-11-18

**Authors:** Ranran Cai, Yunyan Yao, Peng Lv, Yang Ma, Wenyu Xing, Boning Li, Yuan Ji, Huibin Zhou, Chenghao Shen, Shuang Jia, X. C. Xie, Igor Žutić, Qing-Feng Sun, Wei Han

**Affiliations:** 1 International Center for Quantum Materials, School of Physics, Peking University, 100871 Beijing, P. R. China; 2 Collaborative Innovation Center of Quantum Matter, 100871 Beijing, P. R. China; 3 Department of Physics, Wuhan University of Technology, 430070 Wuhan, China; 4 Department of Physics, University at Buffalo, State University of New York, Buffalo, NY 14260 USA; 5 CAS Center for Excellence in Topological Quantum Computation, University of Chinese Academy of Sciences, 100190 Beijing, P. R. China; 6 Beijing Academy of Quantum Information Sciences, 100193 Beijing, P. R. China

**Keywords:** Spintronics, Superconducting properties and materials, Surfaces, interfaces and thin films

## Abstract

Fundamental symmetry breaking and relativistic spin–orbit coupling give rise to fascinating phenomena in quantum materials. Of particular interest are the interfaces between ferromagnets and common s-wave superconductors, where the emergent spin-orbit fields support elusive spin-triplet superconductivity, crucial for superconducting spintronics and topologically-protected Majorana bound states. Here, we report the observation of large magnetoresistances at the interface between a quasi-two-dimensional van der Waals ferromagnet Fe_0.29_TaS_2_ and a conventional *s*-wave superconductor NbN, which provides the possible experimental evidence for the spin-triplet Andreev reflection and induced spin-triplet superconductivity at ferromagnet/superconductor interface arising from Rashba spin-orbit coupling. The temperature, voltage, and interfacial barrier dependences of the magnetoresistance further support the induced spin-triplet superconductivity and spin-triplet Andreev reflection. This discovery, together with the impressive advances in two-dimensional van der Waals ferromagnets, opens an important opportunity to design and probe superconducting interfaces with exotic properties.

## Abstract (web summary)

The interfaces between ferromagnets and superconductors receive many attentions due to emergent relativistic spin-orbit coupling. Here, the authors provide possible evidence for spin triplet Andreev reflection at the interface between a van der Waals ferromagnet Fe_0.29_TaS_2_ and a s-wave superconductor NbN.

## Introduction

Fundamental symmetry breaking and relativistic spin–orbit coupling give rise to interesting phenomena in quantum materials^1,2^. For over 60 years, the interplay between ferromagnetism and superconductivity, has offered a wealth of intriguing phenomena in ferromagnet (FM)/superconductor (SC) heterostructures^3–6^. However, to overcome a strong suppression of spin-singlet superconductivity by the FM’s exchange field the platforms supporting spin-triplet pairing are sought. They are desirable for dissipationless spin currents in superconducting spintronics^5–7^, and probing quantum materials^8^, as well as for realizing elusive Majorana bound states to implement topological quantum computing^9,10^. The common expectation that spin-triplet pairing in superconducting spintronics requires complex FM multilayers, typically relying on noncollinear/spiral magnetization or half metals^3–6,11^.

Here, we report the possible experimental evidence for the spin-triplet Andreev reflection and induced spin-triplet superconductivity at the interface of a quasi-2D van der Waals (vdW) FM and a conventional *s*-wave SC with Rashba spin–orbit coupling (SOC). Such vdW heterostructures offer a great versatility in exploring the interplay between ferromagnetism and superconductivity, beyond the lattice-matching constraints of all-epitaxial FM/SC heterostructures^12^. Our results pave the way for future studies on spin-triplet superconductivity^13,14^ and the formation on Majorana bound states^9,10^, as well as many normal-state spintronic applications^15^.

## Results and discussion

### Spin-triplet Andreev reflection and spin-triplet MR

**Fig. 1:**
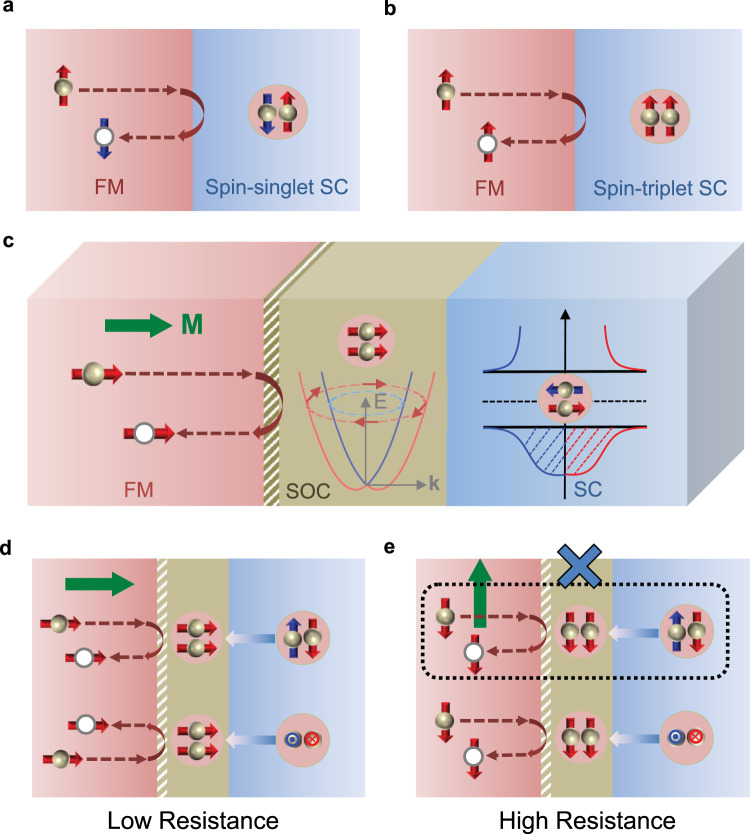
Schematic of the spin-triplet Andreev reflection at FM/SC interface. **a** Conventional Andreev reflection at the FM/spin-singlet SC interface. **b** The spin-triplet Andreev reflection at the FM/spin-triplet SC interface. **c** Schematic of the spin-triplet Andreev reflection resulting from Rashba SOC at the interface between a FM and a conventional *s*-wave SC. The arrows in Rashba SOC band indicate spin-momentum locking and the red arrows represent the spin-polarization direction of equal-spin-triplet pairs. **d**, **e** Anisotropic spin-triplet Andreev reflection at the FM/SC interface and the low/high interfacial resistance states that depend on the FM magnetization direction, **M** (green arrow). Red arrows at the interface denote the spin direction of equal-spin-triplet pairs. For **M** along the interface the spin-triplet Andreev reflection can be suppressed.

In contrast to the conventional Andreev reflection at the FM/SC interface (Fig. 1a), an incident spin-up electron forms a spin-singlet Cooper pair in the ordinary SC with a reflected spin-down hole in the FM, spin-triplet Andreev reflection generates the spin-up hole with an injection of an equal-spin triplet Cooper pair in the spin-triplet SC (Fig. 1b). Due to Rashba SOC^16,17^, spin-rotation symmetry is broken for the superconducting pairing (Fig. 1c), which acts as a spin-mixing described in conventional FM/SC heterostructures^5,6^. The broken spin-rotation symmetry leads to the spin-singlet paring (*m* = 0, *S* = 0) (*S* is the total spin quantum number, and *m* is magnetic quantum number) with an unpolarized spin-triplet component (*m* = 0, *S* = 1)^18^. The spin-triplet component results in the interface spin-triplet Andreev reflection at the FM/SC interface which is highly anisotropic (Supplementary Note 1 and Supplementary Fig. 1), depending on the relative orientation between the magnetization (**M**) in the FM and the interfacial spin–orbit field^14,19^. **M** sets the spin-quantization axis, and unpolarized spin-triplet component (*m* = 0, *S* = 1) is projected onto the spin-quantization axis to generate the equal-spin-triplet component (*m* = 1, *S* = 1), which can be considered as a spin-rotation process^5^. For example, for FM magnetization along *z* axis (perpendicular to the interface), unpolarized spin-triplet Cooper pairs component $\big(|S=1,{S}_{{{{{{\rm{y}}}}}}}=0\rangle \,{{{{{\rm{and}}}}}}\,|S=1,{S}_{{{{{{\rm{x}}}}}}}=0\rangle$ can be projected to the spin quantization axis as $|S=1,{S}_{{{{{{\rm{z}}}}}}}=1{\rangle }$ due to the spin rotation process (${S}_{{{{{{\mathrm{y}}}}}}}$, ${{S}}_{{{{{{\mathrm{x}}}}}}}$, and ${{S}}_{{{{{{\mathrm{z}}}}}}}$ are the spin quantum numbers along *y*, *x*, and *z* direction, respectively). Thus, both the ${|S}=1,{S}_{{{{{{\mathrm{y}}}}}}}=0{\rangle}$ and ${|S}=1,{S}_{{{{{{\mathrm{x}}}}}}}=0{\rangle}$ components will contribute to the interface conductance when **M** is perpendicular to interface, as illustrated in Fig. 1d. On the other hand, when the **M** is parallel to the interface along *y* direction, the equal-spin triplet Cooper pairs ($|S=1,{S}_{{{{{{\mathrm{y}}}}}}}=1{{\rangle }}$) can only be projected from unpolarized spin-triplet pairing component $|S=1,{S}_{{{{{{\mathrm{x}}}}}}}=0{\rangle }$, since $\left[{S}_{{{{{{\mathrm{x}}}}}}},{S}_{{{{{{\mathrm{y}}}}}}}\,\right]\ne\, 0$. Consequently, spin-triplet Andreev reflection conductance channel is suppressed when **M** is parallel to interface, as illustrated in Fig. 1e. As a result of the anisotropic spin-triplet Andreev reflection processes, there is a low-resistance (high-resistance) state for **M** out-of-plane (in-plane) (Fig. 1d, e). Hence, the spin-triplet Andreev reflection can lead to the tunneling anisotropic magnetoresistance (MR) at the FM/SC interface, a proposed hallmark of the interfacial SOC and spin-triplet superconductivity in FM/SC heterostructures^14,19^.

**Fig. 2:**
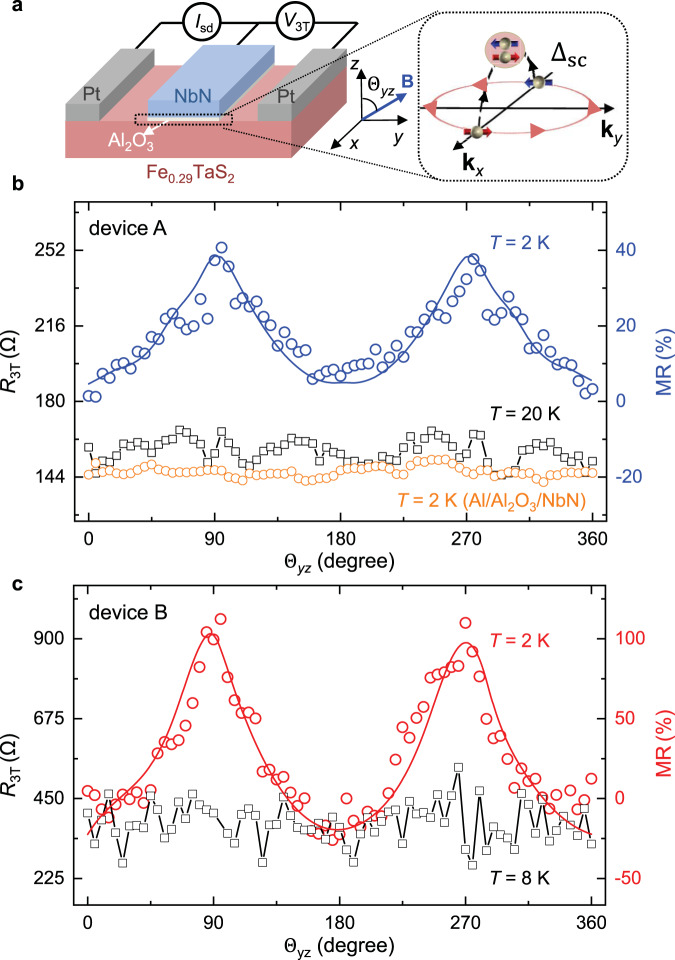
Large magnetoresistance of the quasi-2D vdW Fe_0.29_TaS_2_/SC junction. **a** Illustration of the quasi-2D vdW Fe_0.29_TaS_2_/SC MR device and the measurement geometry. The right panel shows the schematic of the spin-triplet pairing component resulting from Rashba SOC at the FM/SC interface. **b** The interfacial resistance (*R*_3T_ *= V*_3T_/*I*_sd_) and MR ratio as a function of the magnetic field angle measured on the typical quasi-2D vdW Fe_0.29_TaS_2_/SC device (device A) under **B** = 9 T. The orange curve represents the resistance measured on a typical control device (Al/Al_2_O_3_/NbN) under **B** = 9 T. **c** The interfacial resistance and MR ratio as a function of the magnetic field angle on device B under **B** = 9 T. The solid lines in **b** and **c** are guides to the eye.

To experimentally probe the anisotropic spin-triplet Andreev reflection and spin-triplet MR, we fabricate the FM/SC devices (see “Methods” section for details), which consist of a quasi-2D vdW Fe_0.29_TaS_2_ flake, several *s*-wave superconducting NbN electrodes, and two normal metal Pt electrodes (Fig. 2a and Supplementary Fig. 2). At the interface between the quasi-2D vdW Fe_0.29_TaS_2_ flake and *s*-wave NbN electrode, the Cooper pairing consists of both spin-singlet (*m* = 0, *S* = 0) and spin-triplet components (*m* = 0, *S* = 1) due to the spin-rotation symmetry breaking by the interfacial Rashba SOC (right panel of Fig. 2a). The superconducting critical temperature of the NbN electrode is *T*_SC_ ~ 12.5 K (Supplementary Fig. 3a) characterized by standard four-probe electrical measurement. Fe_0.29_TaS_2_ flakes are typical quasi-2D vdW FM, with a Curie temperature, *T*_Curie_, ~ 90 K, characterized by anomalous Hall effect (Supplementary Fig. 4)^20^. The magnetic easy axis is perpendicular to the sample plane, and **M** of Fe_0.29_TaS_2_ can be controlled by a large external magnetic field (**B**) (Supplementary Note 2 and Supplementary Fig. 5). For an in-plane **B** = 9 T, **M** is almost in plane, 83° from the *z* direction. Under **B** = 9 T, the current–voltage characteristics of the NbN electrode are measured, with critical currents of ~ 50 μA at *T* = 2 K (Supplementary Fig. 4b). Typical d*I/*d*V* curves of the Fe_0.29_TaS_2_/SC junctions as a function of *T* and **B** are shown in Supplementary Note 3 and Supplementary Fig. 6.

To characterize the expected MR arising from anisotropic spin-triplet Andreev reflection, the interfacial resistance between the quasi-2D vdW FM Fe_0.29_TaS_2_ and SC electrode is measured using the three-terminal geometry (Fig. 2a and see “Methods” section). Figure 2b shows the typical MR curve (blue) measured (device A; Supplementary Fig. 2) as a function of the magnetic field angle in the *yz* plane (${\Theta }_{{{{{{\rm{yz}}}}}}}$) at *T* = 2 K and **B** = 9 T. The observed MR shows a strong correlation with **B**-controlled **M** (Supplementary Fig. 7). In contrast to this large MR at *T* = 2 K, the normal-state interfacial resistance exhibits little variation at *T* = 20 K. A possible important contribution of vortices in type-II SC to the observed MR has been ruled out from our control measurements on normal metal/SC heterostructures at *T* = 2 K (orange curve in Fig. 2b and Supplementary Note 4 and Supplementary Fig. 8). We have also fabricated the control devices of Fe_0.29_TaS_2_/Al_2_O_3_/normal metal (Al), where no MR could be observed at *T* = 2 K (Supplementary Fig. 9), which further indicates the critical role of SC for the observed MR. Furthermore, the *π*-periodic oscillation further supports that the observed MR results from the anisotropic feature of spin-triplet Andreev reflection at the interface with Rashba SOC^14^. Figure 2c shows the MR results measured on the device B as a function of ${\Theta }_{{{{{{\rm{yz}}}}}}}$ at *T* = 2 K and **B** = 9 T. The MR ratio can be defined as:

1 $${{{{{\rm{MR}}}}}}({\Theta }_{{{{{{\rm{yz}}}}}}})\,=\,\frac{R({\Theta }_{{{{{{\rm{yz}}}}}}})\,-\,R({\Theta }_{{{{{{\rm{yz}}}}}}}\,=\,0)}{R({\Theta }_{{{{{{\rm{yz}}}}}}}\,=\,0)}\times 100 \%.$$

The $R({\Theta }_{{{{{{\rm{yz}}}}}}}=0)$ and $R({\Theta }_{{{{{{\rm{yz}}}}}}}=90)$ are the interfacial resistances for magnetic field that is perpendicular and parallel (along *z* and *y* directions in Fig. 2a) to the FM/SC interface, respectively. Interestingly, the observed MR ratio is ~ 37 ± 2% for device A, and ~ 103 ± 4% for device B, which are much larger than previous reports on the tunneling anisotropic MR in FM/semiconductor heterostructures arising from the Rashba and Dresselhaus SOC^16,17^.

### Temperature evolution of spin-triplet MR

**Fig. 3:**
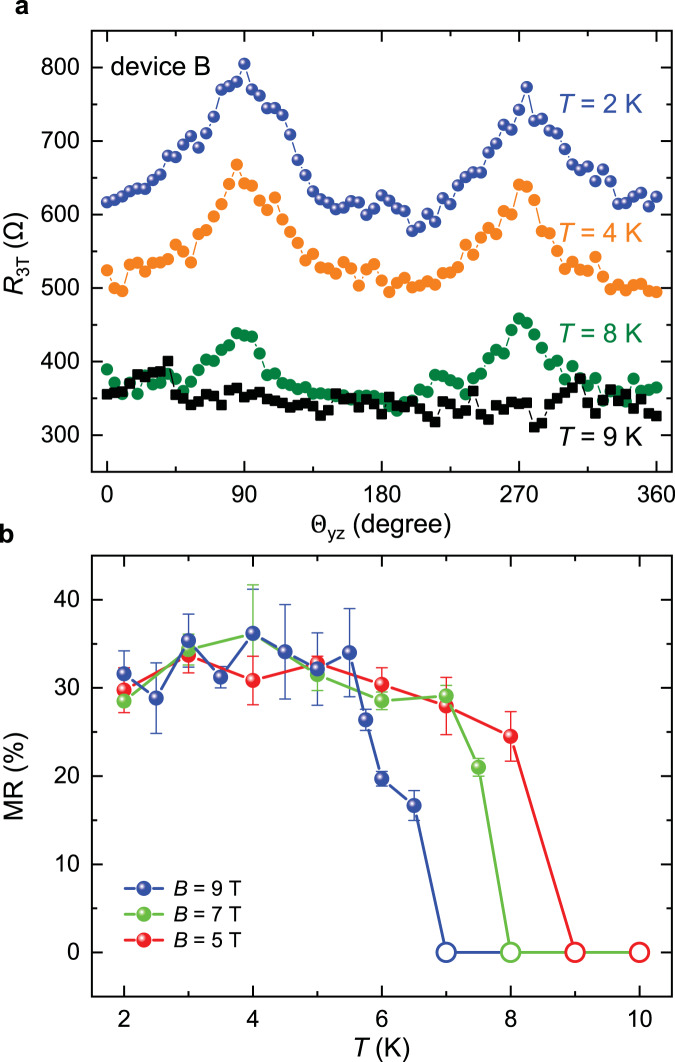
The temperature dependence of MR at Fe_0.29_TaS_2_/SC interface. **a** The interfacial resistance as a function of $\Theta$_yz_ measured on device B at *T* = 2 K (blue), 4 K (yellow) 8 K (olive), and 9 K (black), respectively. These results were obtained under **B** = 5 T and *V*_bias_ = 1 mV, which correspond to *V*_3T_ ~ 0.40 mV for *T* = 2 and 4 K, and *V*_3T_ ~ 0.25 mV for *T* = 8 and 9 K. **b** The temperature dependence of MR ratio of device B at **B** = 9 T, 7 T, and 5 T, respectively. The error bars correspond to one standard deviation. The open circles represent the absence of obvious MR.

Next, we investigate the temperature evolution of the MR to distinguish the contributions from the spin-triplet Andreev reflection and spin-dependent scattering by Bogoliubov quasiparticles under large magnetic field. Figure 3a shows the MR (${\Theta }_{{{{{{\rm{yz}}}}}}}$) for device B at *T* = 2, 4, 8, and 9 K, respectively, under the magnetic field of **B** = 5 T. Figure 3b summarizes temperature dependence of the MR ratio for device B measured at **B** = 9, 7, and 5 T, respectively. The MR appears for *T* < *T*_C_, and starts to saturate below the temperature of ~5 K. The MR is no longer observable for *T* ~ *T*_C_ at **B** = 9, 7, and 5 T (Supplementary Fig. 10). Clearly, there is no enhancement or any anomaly of the MR observed at the temperature slightly below *T*_C_, which further confirms that contribution from spin-dependent scattering by Bogoliubov quasiparticles is negligible^21,22^.

### Voltage dependence of spin-triplet MR

**Fig. 4:**
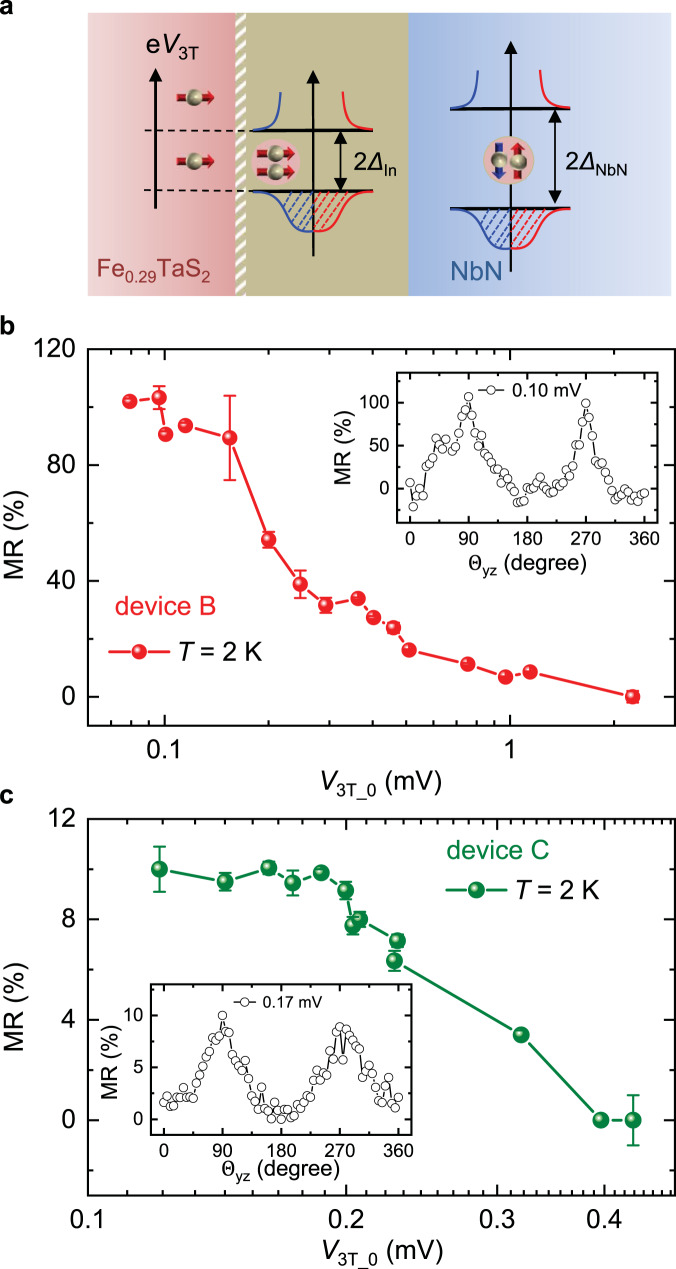
The voltage dependence of MR at Fe_0.29_TaS_2_/SC interface. **a** Schematic of the incident spin-polarized electrons with chemical potentials inside and above the interface spin-triplet superconducting energy gap. Δ_In_ and Δ_NbN_ indicate the superconducting energy gaps of the interface SC and the bulk NbN. **b** The voltage dependence (*V*_3T_0_) of the MR ratio of device B measured at *T* = 2 K and **B** = 9 T. *V*_3T_0_ represents *V*_3T_ when an applied magnetic field is perpendicular to the FM/SC interface. The error bars correspond to one standard deviation. Inset: The typical MR curve at *V*_3T_0_ = 0.10 mV. **c** The voltage dependence (*V*_3T_0_) of the MR ratio of device C measured at *T* = 2 K and **B** = 9 T. The error bars correspond to one standard deviation. Inset: The typical MR curve at *V*_3T_0_ = 0.17 mV.

To further investigate the MR at the quasi-2D vdW FM Fe_0.29_TaS_2_/SC interface, we systematically vary the bias voltage (*V*_bias_), which also affect the junction voltage (*V*_3T_) across the interface. At the interface, the induced SC energy gap (Δ_In_) by SC proximity effect with spin-triplet component is smaller compared to the SC gap (Δ_NbN_) of bulk NbN electrode, as illustrated in Fig. 4a. When the potential (*eV*_3T_) of the incoming electrons is considerably smaller than the interface spin-triplet superconducting energy gap (Δ_In_) (Fig. 4a), the charge transport channel is dominated by the anisotropic spin-triplet Andreev reflection. Hence, the spin-triplet MR exhibits little variation with the e*V*_3T_ within the Δ_In_. As the *V*_3T_ increases, other isotropic transport processes, such as electron-like and hole-like tunneling transmissions^14^, also contribute to the interface conductance. As these transport processes are **M**-independent, the spin-triplet MR ratio is expected to decrease significantly. Since the change of *V*_3T_ is much smaller than *V*_bias_ during the rotation of the external magnetic field, the junction voltage for ${\Theta }_{{{{{{\rm{yz}}}}}}}\,=\,0$ (*V*_3T_0_) is used to qualitatively show the interface voltage dependence of the spin-triplet MR. Figure 4b, c summarize these results measured on devices B and C. For small *V*_3T_0_, the MR exhibit little variation as the voltage changes. However, when *V*_3T_0_ is higher than a critical value, MR strongly decreases as *V*_3T_ increases. The critical junction voltage is obtained to be ~0.15 mV (~0.2 mV) for device B (C). We note that at 2 K the thermal energy is ${k}_{{{{{{\rm{B}}}}}}}T$ ~ 0.17 meV, comparable to the critical electron potential from the bias-dependent results. Therefore, an accurate value of the proximity-induced superconducting gap is not able to be clearly resolved here, which will need future studies. Additionally, the bias dependence of the spin-triplet MR further confirms that the observed MR is correlated to the sub-gap properties, and is completely different form **B**-induced spin-splitting density of states at the gap edges of SC electrodes^23^.

### Interface barrier dependence of spin-triplet MR

**Fig. 5:**
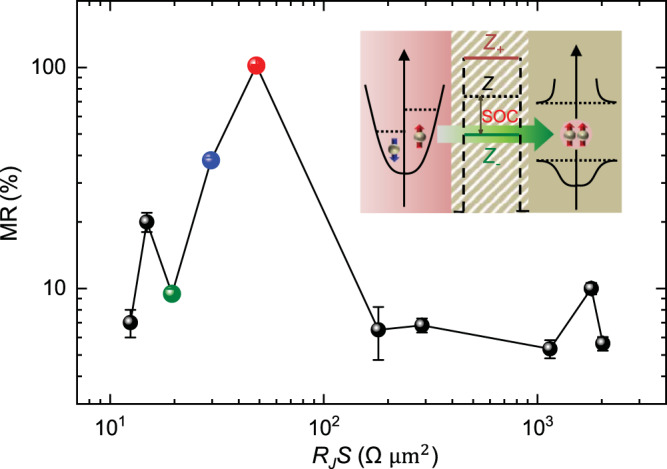
The interface barrier dependence of MR at Fe_0.29_TaS_2_/SC interface. The MR ratio as a function of the interface resistance area product (*R*_J_S) measured on various devices in the low voltage bias region. Inset: Schematic of the incident spin-polarized electrons into the interfacial spin-triplet SC via interface barrier with Rashba SOC. The Rashba SOC modifies the interface barrier strength (*Z*) to be ${Z}_{\pm }=Z\pm {\bar{\gamma }k}_{\parallel }$, where $\bar{\gamma }$ is the SOC parameter and ${k}_{\parallel }$ is the in-plane wave vector^25^. The blue, red, and green dots represent the MR of devices A, B, and C, respectively. The error bars correspond to one standard deviation.

As the spin-triplet Andreev reflection depends strongly on the FM and SC wave-function overlap, it is expected that the dimensionless interface barrier strength (*Z*) plays an important role in the spin-triplet MR^24,25^. To explore the influence of interface barrier strength on the observed spin-triplet MR, we investigate more than dozen devices that are fabricated with Al_2_O_3_ layer of different thickness (~1–2.5 nm) between the quasi-2D vdW FM Fe_0.29_TaS_2_ and NbN SC electrodes. This process leads to a large range of interface resistance area product (*R*_J_*S*) from ~10 to ~2000 Ω μm^2^, resulting in the FM/SC heterostructures with very different *Z*-values. Figure 5 shows the measured MR ratio as a function of the *R*_J_*S* at *T* = 2 K and **B** = 9 T (Note: the MR is not observable for very large *R*_J_*S* and not plotted in this figure). The largest MR is observed with *R*_J_*S* ~ 48.4 Ω μm^2^. The strong correlation of the MR ratio and *R*_J_*S* reveals the important role of the *Z*-value in the spin-triplet MR.

This surprising nonmonotonic MR dependence on *R*_J_*S* agrees well with the theoretical expectations^14,25^. The effective barrier strength is modified by SOC and depends on the helicity (outer/inner Rashba bands, Fig. 1b), ${Z}_{\pm }=Z\pm {\bar{\gamma }k}_{\parallel }$, where $\bar{\gamma }$ is the SOC parameter^25^ and ${k}_{\parallel }$ is the component of the wave vector along the interface (Fig. 5 inset). At zero ${k}_{\parallel }$, the vanishing of Rashba SOC does not support spin-triplet component. At nonzero ${{k}}_{\parallel },$ increasing $Z$ can reduce ${{|Z}}_{+}|$ or ${{|Z}}_{-}{|}$ and thus enhance such a transmission for a given helicity. For much larger $Z$, all of the conduction channels, including spin-triplet Andreev reflection, are suppressed due to the low interface transparency. As a result, the spin-triplet Andreev reflection and spin-triplet MR will also be nonmonotonic in *Z*. Taken together, the observed nonmonotonic MR dependence with *R*_*J*_*S* (Fig. 5) and MR decrease with *T* or an applied voltage (Figs. 2–4) are all experimental evidence for the spin-triplet Andreev reflection in our vdW heterostructures. We note that the spin-triplet MR theory is developed using an idealized model of ballistic systems^14,25^, the role of disorder, which could induce reflectionless tunneling, is expected to reduce the MR amplitude. To the best of our understanding, the spin-triplet Andreev reflection is the major cause for the observation of large MR up to ~103 ± 4%, and can qualitatively explain the bias and temperature dependence of the MR. Given the growing interest in systems that could support spin-triplet superconductivity, in the future studies, it would be important to generalize our description and also include the effects of disorder and diffusive transport on spin-triplet MR.

### Summary and outlook

Our experimental obervation of a large tunneling anisotropic MR in quasi-2D vdW FM/s-wave SC heterostructures up to ~103 ± 4% is already promising for spintronic applications and much larger than for the normal-state transport in previously measured heterostructures with a single FM layer^16,17^. More importantly, this result also reveals an emergent spin-triplet superconductivity which, through spin-triplet Andreev reflection, is a sensitive probe of interfacial Rashba SOC. With the advances towards high-quality vdW heterostructures, we anticipate that the magnitude of such spin-triplet MR can be further enhanced and strongly modulated using different 2D vdW FMs due to their highly-tunable Rashba SOC by electric fields^26–29^. This tantalizing opportunity to implement FM/SC heterostructures to design and probe interfacial SOC offers an important boost for superconducting spintronics^3–6,30,31^ and Majorana bounds states^9,32^. Furthermore, our quasi-2D platform of proximity-induced spin-triplet superconductivity, combined with the gate-controlled 2D vdW ferromagnetism^28,29,33^ could provide tunable magnetic textures to create synthetic SOC^34^ and braid Majorana bound states^35^.

## Methods

### Device fabrication

The quasi-2D vdW Fe_0.29_TaS_2_/SC spin-triplet MR devices were fabricated as follows. First, bulk single crystalline Fe_0.29_TaS_2_ were grown by the iodine vapor transport method. Then the quasi-2D vdW Fe_0.29_TaS_2_ flakes were mechanical exfoliated from the bulk single crystal onto the SiO_2_ (~300 nm)/Si substrates^20^. Second, a first-step electron-beam lithography was used to define the SC electrodes on the quasi-2D vdW Fe_0.29_TaS_2_ flakes. The SC electrodes consist of ~5 nm thick Nb and ~60 nm thick NbN, which were grown in a DC magneton sputtering system with a base pressure of ~1.2 × 10^−4^ Pa. Prior to the growth of SC electrodes, a thin Al_2_O_3_ layer (~1–2.5 nm) is deposited as the barrier to tune the interface coupling strength between the quasi-2D vdW Fe_0.29_TaS_2_ flakes and the SC electrodes. The Al_2_O_3_ layer was grown by DC magnetron sputtering with Al target under the oxygen atmosphere. Then, a second-step electron-beam lithography was used to define the two normal Pt electrodes (~80 nm) on the quasi-2D vdW Fe_0.29_TaS_2_ flakes. The Pt electrodes were deposited by RF magneton sputtering in a system with a base pressure lower than 6.5 × 10^−4^ Pa. The optical images of three typical devices (A, B, and C) are shown in Fig. S2.

### Spin-triplet MR measurement

The MR measurement of the quasi-2D vdW Fe_0.29_TaS_2_/SC devices was performed in a Physical Properties Measurement System (PPMS; Quantum Design). A bias (*V*_bias_) was applied between the SC electrode and one normal Pt electrode using a Keithley K2400, the source–drain current (*I*_sd_) was measured using the same K2400, and the voltage (*V*_3T_) between the SC electrode and the other Pt electrode was measured using a Keithley 2002. The interfacial resistance was obtained via dividing the three-terminal voltage by the source–drain current (*R*_3T_ = *V*_3T_/*I*_sd_). During the measurement of each spin-triplet MR curve, the quasi-2D vdW Fe_0.29_TaS_2_/SC device was rotated from 0 to 360 degrees under the external static magnetic field in the PPMS.

## Supplementary information

Supplementary Information

## Data Availability

The data that support the findings of this study are available from the corresponding authors upon request.
