# Supplementary material for: Evidence for anisotropic spin-triplet Andreev reflection at the 2D van der Waals ferromagnet/superconductor interface
Source: Nat Commun. 2021 Nov 18;12:6725. doi: 10.1038/s41467-021-27041-w (PMC8602320; doi:10.1038/s41467-021-27041-w)
Supplement: Supplementary file 1 — Supplementary Information [file 41467_2021_27041_MOESM1_ESM.pdf]

Supplementary Information for

Evidence for spin-triplet Andreev reflection at the 2D van der Waals ferromagnet/superconductor interface

Ranran Cai[1,2†], Yunyan Yao[1,2†], Peng Lv[3], Yang Ma[1,2], Wenyu Xing[1,2], Boning Li[1,2], Yuan Ji[1,2] Huibin Zhou[1,2], Chenghao Shen[4], Shuang Jia[1,2,5,6], X. C. Xie[1,2,5,6], Igor Zutic[4], Qing-Feng Sun[1,2,5,6] & Wei Han[1,2]*

[1]International Center for Quantum Materials, School of Physics, Peking University, Beijing 100871, P. R. China

[2]Collaborative Innovation Center of Quantum Matter, Beijing 100871, P. R. China

[3]Department of Physics, Wuhan University of Technology, Wuhan 430070, China

[4]Department of Physics, University at Buffalo, State University of New York, Buffalo, New York 14260, USA

[5]CAS Center for Excellence in Topological Quantum Computation, University of Chinese Academy of Sciences, Beijing 100190, P. R. China

[6]Beijing Academy of Quantum Information Sciences, Beijing 100193, P. R. China

[†]These authors contributed equally to the work

*Correspondence to: weihan@pku.edu.cn (W.H.)

This file includes the following:

1) Supplementary Notes 1-4
2) Supplementary References
3) Supplementary Table 1
4) Supplementary Figures 1-10

Supplementary Note 1. Spin-triplet pairings induced by interfacial spin-orbit couplings

Due to the lack of inversion symmetry at the interface between a ferromagnet (FM) and an s-wave superconductor (SC), there are generally two types of spin-orbit coupling (SOC), namely Rashba and Dresselhaus SOC. Close to the interface, the spin-momentum locking property of SOC breaks the spin-rotation symmetry, leading to spin-triplet Cooper pairs and spin-triplet Andreev reflection at the FM/SC interface. In this section, we calculate the correlation functions of the superconductor with both Rashba and Dresselhaus SOC. Consider the normal-state Hamiltonian of the superconductor as follows[1],

$$h(\mathbf{k}) = \sum_{\mathbf{k},s} \varepsilon_{\mathbf{k}}\, c_{\mathbf{k}s}^{\dagger} c_{\mathbf{k}s} + \sum_{\mathbf{k},ss'} \left[\alpha_R(k_y\sigma_x - k_x\sigma_y) + \alpha_D(k_x\sigma_x - k_y\sigma_y)\right]_{ss'} c_{\mathbf{k}s}^{\dagger} c_{\mathbf{k}s'}, \tag{1}$$

where $\varepsilon_{\mathbf{k}} = \frac{\hbar^2\mathbf{k}^2}{2m_S} - \mu_S$ is the single-electron kinetic energy measured from the chemical potential μ_S, m_S is the electron mass in SC and $\hbar$ is the reduced Plank constant, $c_{\mathbf{k}s}^{\dagger}(c_{\mathbf{k}s'})$ is the creation (annihilation) operator of electron, $s, s' = \uparrow, \downarrow$ are the spin indices, α_R and α_D are the Rashba and Dresselhaus SOC strength parameters, respectively, $\mathbf{k} = (k_x, k_y, k_z)$ is the three-dimensional electron wave vector, and (σ_x, σ_y) are the Pauli matrices in the *x*-*y* plane. Assuming the usual spin-singlet s-wave paring, the mean-field Bogoliubov-de Gennes (BdG) Hamiltonian of Supplementary Eq. (1) in the Nambu basis $(c_{\mathbf{k}\uparrow}, c_{\mathbf{k}\downarrow}, c_{-\mathbf{k}\uparrow}^{\dagger}, c_{-\mathbf{k}\downarrow}^{\dagger})$ can be written as

$$H(\mathbf{k}) = \begin{pmatrix} h(\mathbf{k}) & i\sigma_y\Delta \\ -i\sigma_y\Delta & -h^*(-\mathbf{k}) \end{pmatrix}. \tag{2}$$

The in-plane SOC breaks the spin-rotation symmetry of $h(\mathbf{k})$, and then the *s*-wave pairing matrix $i\sigma_y\Delta$ in Supplementary Eq. (2) can induce spin-triplet Cooper pairs. This can be shown by

studying the pairing symmetry. After solving the standard Gor'kov equations[2,3], the corresponding pairing correlations can be written as

$$\mathbf{F}(\mathbf{k},E) = \Delta[d_0(\mathbf{k},E)\sigma_0 + \mathbf{d}(\mathbf{k},E)\cdot\boldsymbol{\sigma}]i\sigma_y. \tag{3}$$

where $d_0(\mathbf{k},E) = F_+(\mathbf{k},E)$ characterizes the spin-singlet part, $\mathbf{d}(\mathbf{k},E) = F_-(\mathbf{k},E)\hat{\mathbf{g}}_\mathbf{k}$ characterizes the spin-triplet part, E is the energy eigenvalue, $\mathbf{g}_\mathbf{k} = (\alpha_R k_y + \alpha_D k_x, -(\alpha_R k_x + \alpha_D k_y), 0)$ is the SOC field with unit vector $\hat{\mathbf{g}}_\mathbf{k} = \mathbf{g}_\mathbf{k}/|\mathbf{g}_\mathbf{k}|$, and

$$F_\pm(\mathbf{k},E) = \frac{1}{2}\left[\frac{1}{E^2-(\varepsilon_\mathbf{k}+|\mathbf{g}_\mathbf{k}|)^2-\Delta^2} \pm \frac{1}{E^2-(\varepsilon_\mathbf{k}-|\mathbf{g}_\mathbf{k}|)^2-\Delta^2}\right]. \tag{4}$$

In the absence of the SOC field (i.e., $|\mathbf{g}_\mathbf{k}| = 0$), the spin-triplet part $\mathbf{d}(\mathbf{k},E) = F_-(\mathbf{k},E)\hat{\mathbf{g}}_\mathbf{k}$ vanishes, and the system exhibits conventional s-wave superconductivity. On the other hand, in the presence of the SOC with the nonzero $\mathbf{g}_\mathbf{k}$, the system supports mixed s-wave and p-wave superconductivity, with the spin-triplet part being linearly proportional to $|\mathbf{g}_\mathbf{k}|$ for small SOC field. It is important to note that in the basis where the spin-quantization axis is along the out-of-plane direction (z-direction, see Fig. 2a in the main text), only d_x, d_y components of the spin-triplet part $\mathbf{d}(\mathbf{k},E)$ are nonzero, and all the spin-triplet Cooper pairs are formed by electrons with equal spins (Supplementary Table 1). Hence, besides conventional Andreev reflection, spin-triplet Andreev reflection also occur at the FM/SC interface, as shown in Supplementary Fig. 1a. Here, we emphasize that $\sqrt{d_x^2 + d_y^2}$ is nonzero for arbitrary in-plane wave vector (k_x, k_y), which indicates that the spin-triplet Andreev reflection can occur without any constraint if the spins of the incident electrons are along the out-of-plane direction.

For incident electrons with spins pointing along the *xy* plane, the spin-triplet Andreev reflection can be suppressed for certain in-plane wave vectors. For example, considering the spins

of incident electrons being along the x axis, $\sqrt{d_y^2+d_z^2}=|\alpha_R k_x+\alpha_D k_y|$ can be zero at some special wave vectors (k_x, k_y), leading to the disappearance of the spin-triplet Andreev reflection. To see this more clearly, we introduce an angle ϑ to denote the strength ratio between Dresselhaus and Rashba SOC, with $\alpha_R=\gamma\cos\vartheta$, $\alpha_D=\gamma\sin\vartheta$, $\gamma=\sqrt{\alpha_R{}^2+\alpha_D{}^2}$. We also introduce an angle φ to denote the azimuth of in-plane wave vector $k_\parallel$, with $k_x=k_\parallel\cos\varphi$, $k_y=k_\parallel\sin\varphi$, $k_\parallel=\sqrt{k_x^2+k_y^2}$. Then the SOC field $\mathbf{g_k}$ can be simplified to $(k_\parallel\gamma\sin(\varphi+\vartheta), -k_\parallel\gamma\cos(\varphi-\vartheta), 0)$. By defining $\sin\rho=\sin(\varphi+\vartheta)/A$, $\cos\rho=\cos(\varphi-\vartheta)/A$, $A=\sqrt{1+\sin2\vartheta\sin2\varphi}$, the pairing correlations in Supplementary Eq. (S3) can be rewritten as

$$\mathbf{F}(\mathbf{k},E)=\Delta\begin{pmatrix}-ie^{-i\rho}F_- & F_+\\ -F_+ & -ie^{i\rho}F_-\end{pmatrix}. \tag{5}$$

By choosing a new spin-quantization axis in the *x*-*y* plane, the pairing correlations become

$$\tilde{\mathbf{F}}(\mathbf{k},E)=\frac{\Delta}{2}\begin{pmatrix}-ie^{-i\rho}\left[e^{2i(\rho-\phi)}+1\right]F_- & 2F_+ + ie^{i(\phi-\rho)}\left[1-e^{2i(\rho-\phi)}\right]F_-\\ -2F_+ + ie^{i(\phi-\rho)}\left[1-e^{2i(\rho-\phi)}\right]F_- & -ie^{i\rho}\left[e^{2i(\phi-\rho)}+1\right]F_-\end{pmatrix}, \tag{6}$$

where ϕ is the angle between the spin-quantization axis and the x axis. The spin-triplet Andreev reflection arises from the diagonal part of the paring correlations, which vanishes if

$$e^{2i(\rho-\phi)}=-1\Rightarrow\rho=\phi\pm\pi/2\,, \tag{7}$$

and reaches a maximum if

$$e^{2i(\rho-\phi)}=1\Rightarrow\rho=\phi,\phi+\pi\,. \tag{8}$$

Note that the parameter ρ encodes the in-plane wave vector (k_x, k_y) of the spin-polarized electrons, which means that for certain in-plane wave vectors, the spin-triplet Andreev reflection

is strictly prohibited. Compared with the case where the electrons' spin directions are out-of-plane, we can infer that the conductance is weaker when the spin directions of incident electrons are in-plane. As an example, consider the spin direction of incident electrons and the spin-quantization along the x axis (i.e., $\phi = 0$).

In the absence of the Dresselhaus SOC ($\vartheta = 0$), we have $\rho = \varphi$ from the definition of ρ. The minimum condition in Supplementary Eq. (S7) now reduces to

$$\varphi = \pm\pi/2 \Rightarrow k_x = 0, k_y \neq 0 \, , \tag{9}$$

which means for incident electrons with in-plane wave vector $(0, k_y)$, the spin-triplet Andreev reflection is strictly prohibited, since the Cooper pairs are completely formed by electrons with opposite spins, as illustrated in Supplementary Fig. 1b. On the other hand, the maximum condition in Supplementary Eq. (S8) now reduce to

$$\varphi = 0, \pi \Rightarrow k_x \neq 0, k_y = 0 \, , \tag{10}$$

which means for incident electrons with in-plane wave vector $(k_x, 0)$, the spin-triplet Andreev reflection is the most significant as shown in Supplementary Fig. 1c.

Supplementary Note 2. Characterization of quasi-2D vdW FM $Fe_{0.29}TaS_2$ via anomalous Hall effect

The magnetic properties of quasi-2D vdW FM $Fe_{0.29}TaS_2$ are measured via anomalous Hall effect (AHE) on the Hall bar devices (Supplementary Fig. 4 and Fig. 5). The Curie temperature is obtained to be ~ 90 K at the temperature when the anomalous Hall resistance disappears (Supplementary Fig. 4d). As shown in Supplementary Fig. 4c and Supplementary Fig. 5a, the magnetization (**M**) easy axis is perpendicular to the $Fe_{0.29}TaS_2$ plane. The angle between **M** and

the interface normal (θ_M) can be estimated using the evolution of AHE with applied magnetic field, from $\theta_M = \arccos\,(R_{AHE}^{\mathbf{B_{IP}}}/R_{AHE}^{\mathbf{B}_\perp})$, where $\mathbf{B}_{\mathrm{IP}}$ and $\mathbf{B}_\perp$ represent the in-plane and out-of-plane magnetic fields and they label the corresponding AHE resistance. The magnetic field dependence of θ_M is shown in Supplementary Fig. 5b.

Supplementary Fig. 7 shows the MR results as a function of the extenal magnetic field measured on device B. Clearly, similar trends of the MR and θ_M are observed as a function of the magnetic field. This observation suggests that the measured MR is **M**-dependent, which is consistent with the strong anisoptric spin-triplet Andreev refelction at the $Fe_{0.29}TaS_2$/SC interface.

Supplementary Note 3. Bias dependence of the conductance at the $Fe_{0.29}TaS_2$/SC interface.

The interface conductance is characterized by the bias dependence at various temperature and magnetic fields (Supplementary Fig. 6). Around zero bias, the conductance at $T = 2$ K is about 0.8 of the normal state value, G_N, which indicates that the interface barrier has a modest strength, rather than a strong barrier with a low transparency[4]. For such a modest interfacial strength as in our $Fe_{0.29}TaS_2$/SC junctions, the contribution of the Andreev reflection to the zero-bias interface conductance remains significant.

Supplementary Note 4. Control experiments on the vortex-induced anisotropic transport in superconductor heterostructures

In this section, we discuss the role of vortex-induced anisotropic interfacial resistance in the type-II SC heterostructures[5,6]. As it is well known that the formation of vortices in SC is strongly dependent on the direction and amplitude of the external mangetic field[6,7], the vortices in type-II SC might also contribute to the anisotropic transport properties in the FM/SC heterostructures. To

study the role of the vortices and the anisotropic interfacial resistance purely from vortex in the SC NbN electrode, we fabricate control devices that use ~20 nm Al as a NM to replace the quasi-2D vdW FM $Fe_{0.29}TaS_2$. As seen in Fig. S8, The only difference between the spin-triplet MR device (Supplementary Fig. 8a) and control device (Supplementary Fig. 8b) is the bottom layer; NM Al vs. quasi-2D vdW FM $Fe_{0.29}TaS_2$. These two devices are chosen for the comparision due to similar values of the interface resistance area product (R_JS) of 29.7 and 45.6 Ω μm^2 for spin-triplet MR device and control device, respectively.

Using the same measurement geomery and under the same conditions (T = 2 K, **B** = 9 T, and V_{bias} = 1 mV) as the spin-triplet MR device, the angle dependence of the interfacial resistance between the Al electrode and the NbN electrode is measured. Clearly, the vortex-induced MR in control device (red symbols in Supplementary Fig. 8c) is significantly smaller compared to the spin-triplet MR (blue symbols in Supplementary Fig. 8c). Furthermore, the MR in the control device is within the noise level of ~3% (Supplementary Fig. 8d). To rule out any significant contribution from vortex in SC, the control devices with various R_JS have been fabricated and measured. The signals of these control devices are summarized in Supplementary Fig. 8e (red symbols), which are significantly smaller compared to the MR at quasi-2D vdW $Fe_{0.29}TaS_2$/SC interface (blue symbols). To conclude, the contribution of the vortices in type-II SC to the interface transport properties is negligible compared to the spin-triplet MR in our study on the spin-triplet Andreev reflection of the quasi-2D vdW FM $Fe_{0.29}TaS_2$/SC devices.

Supplementary References:

1 Bauer, E. & Sigrist, M. *Non-Centrosymmetric Superconductors: Introduction and Overview*. (Springer Berlin Heidelberg, 2012).

2 Gor'kov, L. P. & Rashba, E. I. Superconducting 2D System with Lifted Spin Degeneracy: Mixed Singlet-Triplet State. *Phys. Rev. Lett.* **87**, 037004, (2001).

3 Zhou, B. T., Yuan, N. F. Q., Jiang, H.-L. & Law, K. T. Ising superconductivity and Majorana fermions in transition-metal dichalcogenides. *Phys. Rev. B* **93**, 180501, (2016).

4 Blonder, G. E., Tinkham, M. & Klapwijk, T. M. Transition from metallic to tunneling regimes in superconducting microconstrictions: Excess current, charge imbalance, and supercurrent conversion. *Phys. Rev. B* **25**, 4515-4532, (1982).

5 Miyoshi, Y., Bugoslavsky, Y. & Cohen, L. F. Andreev reflection spectroscopy of niobium point contacts in a magnetic field. *Phys. Rev. B* **72**, 012502, (2005).

6 Rosenstein, B. & Li, D. Ginzburg-Landau theory of type II superconductors in magnetic field. *Rev. Mod. Phys.* **82**, 109-168, (2010).

7 Tinkham, M. Effect of Fluxoid Quantization on Transitions of Superconducting Films. *Phys. Rev.* **129**, 2413-2422, (1963).

$d_{0,x,y,z}$	pairing symmetry	value	type of AR
d_0	singlet	$1/2\langle c_{k\uparrow}c_{-k\downarrow} - c_{k\downarrow}c_{-k\uparrow}\rangle$	conventional AR
d_x	triplet	$-1/2\langle c_{k\uparrow}c_{-k\uparrow} - c_{k\downarrow}c_{-k\downarrow}\rangle$	spin-triplet AR
d_y	triplet	$-i/2\langle c_{k\uparrow}c_{-k\uparrow} + c_{k\downarrow}c_{-k\downarrow}\rangle$	spin-triplet AR
d_z	triplet	$1/2\langle c_{k\uparrow}c_{-k\downarrow} + c_{k\downarrow}c_{-k\uparrow}\rangle$	conventional AR

Supplementary Table 1: A summary list of the pairing functions of mixed s- and p-wave superconductivity. The spins of incident electrons are chosen to be along the *z* axis. The singlet part d_0 and the triplet part d_z are formed by electrons with opposite spins along the *z* direction, while d_x and d_y are formed by electrons with equal spins. The nonzero d_x or d_y gives rise to unconventional spin-triplet Andreev reflection (AR).

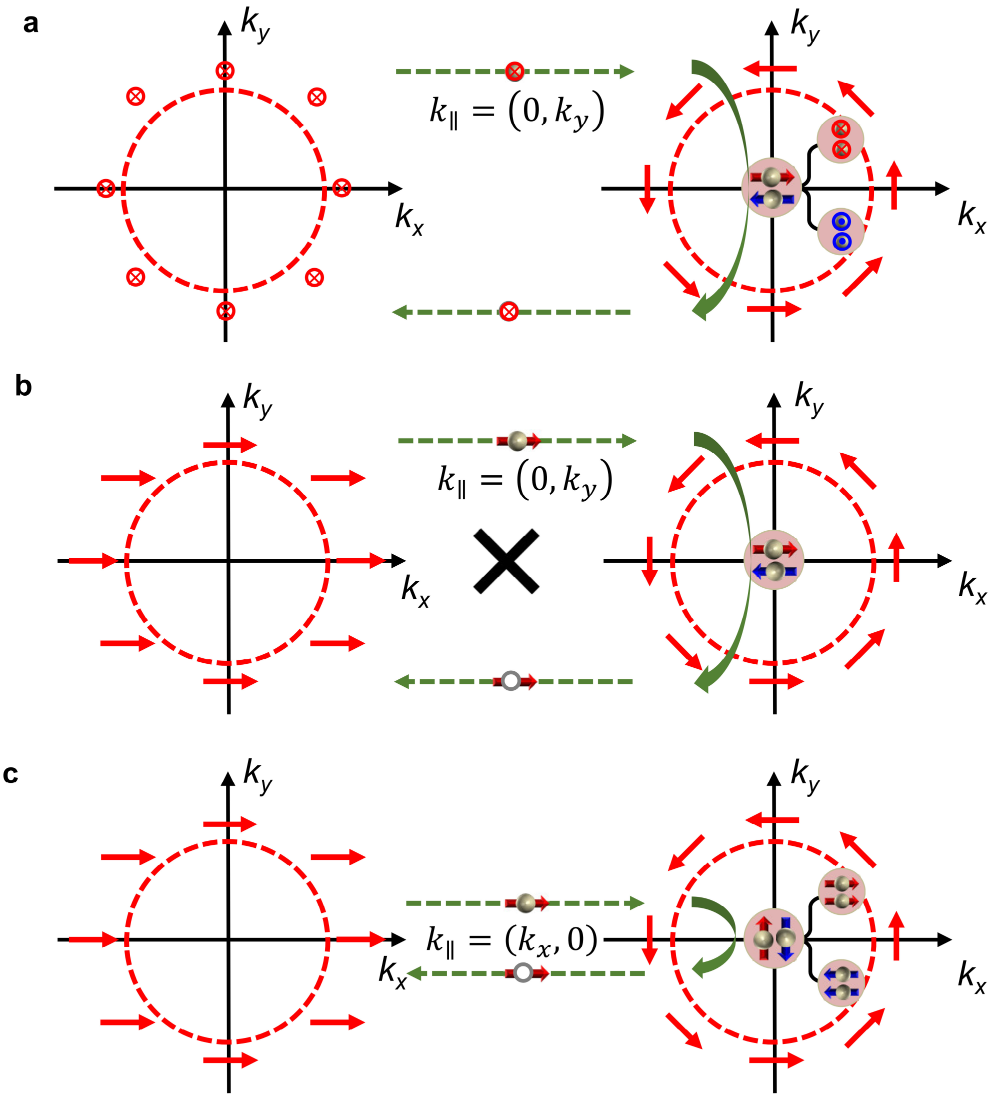

Supplementary Figure 1. The schematic diagram of the spin-triplet Andreev reflection at the FM/SC interface. a, For incident electrons with out-of-plane spins, the spin-triplet Andreev reflection can occur for arbitrary in-plane wave vector ($\mathbf{k}_x$, $\mathbf{k}_y$), due to the formation of equal-spin Cooper pairs. **b, c,** For incident electrons with spins along the *x* axis, the spin-triplet Andreev reflection cannot occur for in-plane wave vector (0, $\mathbf{k}_y$), but is significant for in-plane wave vector ($\mathbf{k}_x$, 0).

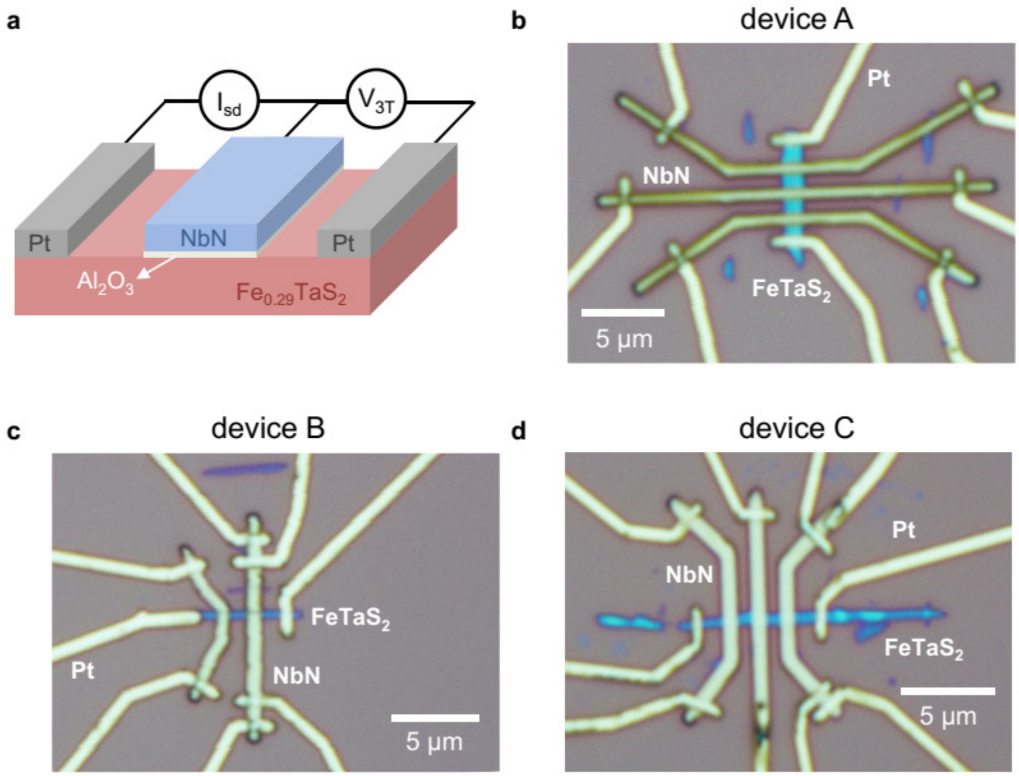

Supplementary Figure 2. The optical images of the representative devices. a, Illustration of the quasi-2D vdW $Fe_{0.29}TaS_2$/SC MR device and the measurement geometry. Between the SC and the $Fe_{0.29}TaS_2$ flake, a thin Al_2O_3 layer (~ 1 - 2.5 nm) is used to tune the interface coupling strength. **b,** The optical image of Device A. Three SC NbN electrodes are fabricated onto the central part of the $Fe_{0.29}TaS_2$ flake, and two normal metal Pt electrodes are contacted on the two ends of the $Fe_{0.29}TaS_2$ flake. The thickness of the $Fe_{0.29}TaS_2$ flake is estimated to be ~ 20 nm. **c,** The optical image of device B. The thickness of the $Fe_{0.29}TaS_2$ flake is estimated to be ~ 15 nm. **d,** The optical image of device C. The thickness of the $Fe_{0.29}TaS_2$ flake is estimated to be ~ 20 nm.

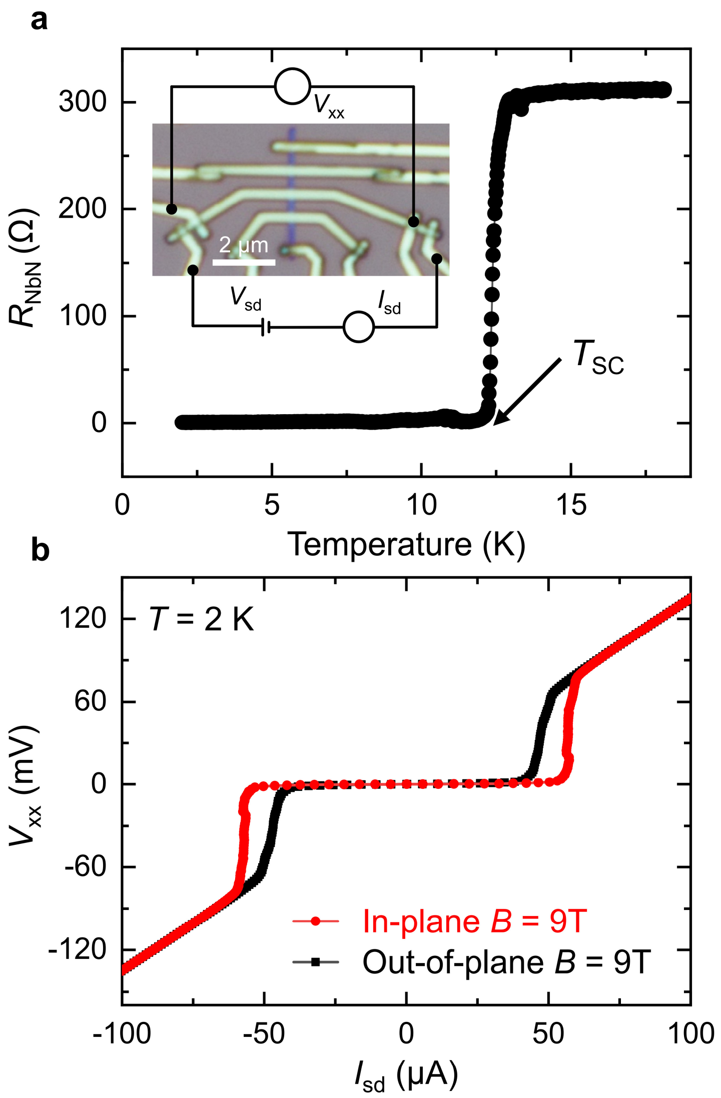

Supplementary Figure 3. Characterization of the SC electrodes. **a**, The resistance of the NbN electrode as a function of the temperature. The superconducting critical temperature (T_{SC}) is determined to be ~ 12.5 K. Inset: The characterization of the SC electrode's resistance on a typical device using the standard four-probe measurement geometry. **b**, The current-voltage characteristics of the NbN electrode measured at $T = 2$ K under the in-plane (red) and out-of-plane (black) magnetic fields ($B = 9$ T), respectively.

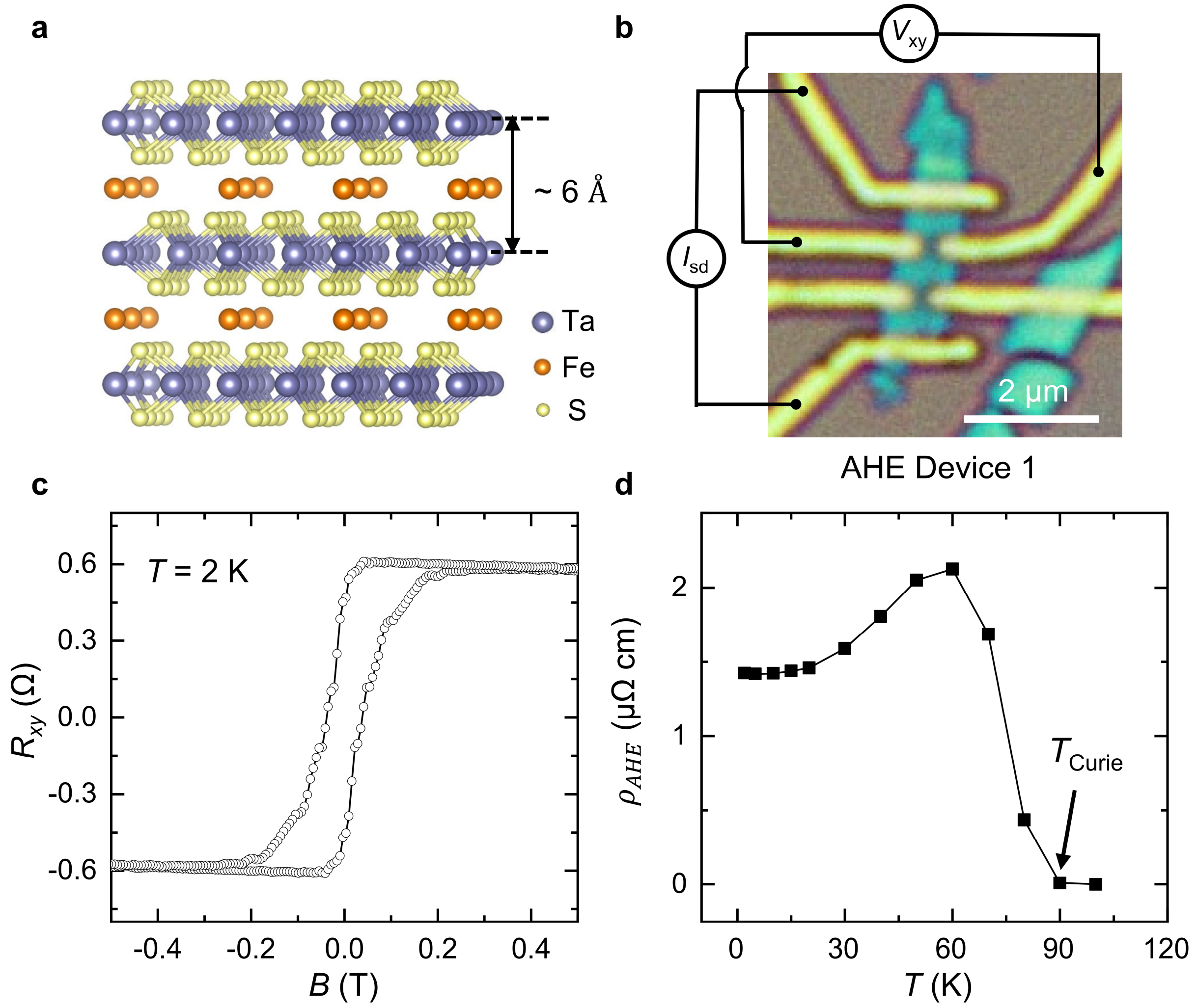

Supplementary Figure 4. Magnetic properties of quasi-2D vdW $Fe_{0.29}TaS_2$. a, The side view of the crystal structure of itinerant quasi-2D vdW FM $Fe_{0.29}TaS_2$ flakes. The Fe atoms are located between the TaS_2 layers, which are stacked together via vdW interaction with an interlayer distance of ~ 6 Å. **b,** The optical image of the $Fe_{0.29}TaS_2$ anomalous Hall effect (AHE) device and the measurement geometry. The thickness of the quasi-2D vdW $Fe_{0.29}TaS_2$ is ~ 14 nm determined via atomic force microscopy. **c**, The transverse resistance (R_{xy}) as a function of the out-of-plane magnetic field measured on the $Fe_{0.29}TaS_2$ AHE device at T = 2 K. **d**, The temperature-dependent anomalous Hall resistivity of the quasi-2D vdW $Fe_{0.29}TaS_2$ flake. The Curie temperature (T_{Curie}) is ~ 90 K, indicated by the black arrow.

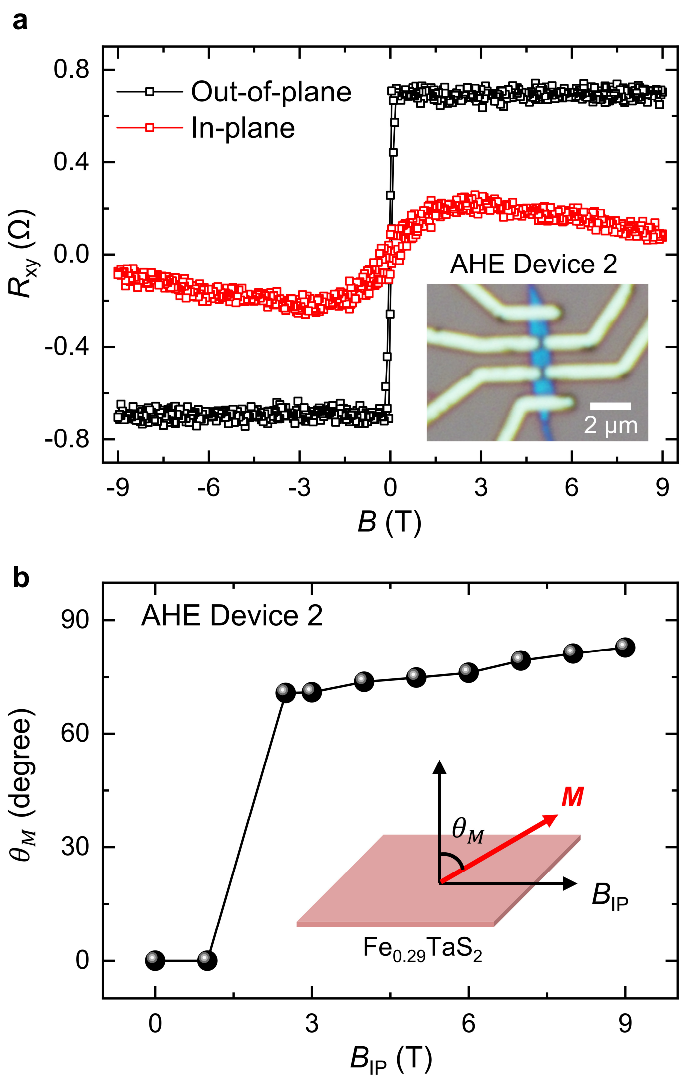

Supplementary Figure 5. Magnetization angle characterization of quasi-2D vdW $Fe_{0.29}TaS_2$ via anomalous Hall effect. a, The transverse resistance (R_{xy}) as a function of the out-of-plane and in-plane magnetic fields measured on the $Fe_{0.29}TaS_2$ AHE device at T = 2 K. Inset: The optical image of the $Fe_{0.29}TaS_2$ AHE device. The thickness of the 2D vdW $Fe_{0.29}TaS_2$ is ~ 20 nm. **b**, The magnetization angle as a function of the in-plane magnetic field ($\mathbf{B}_{IP}$). Inset: The schematic of θ_M under applied $\mathbf{B}_{IP}$.

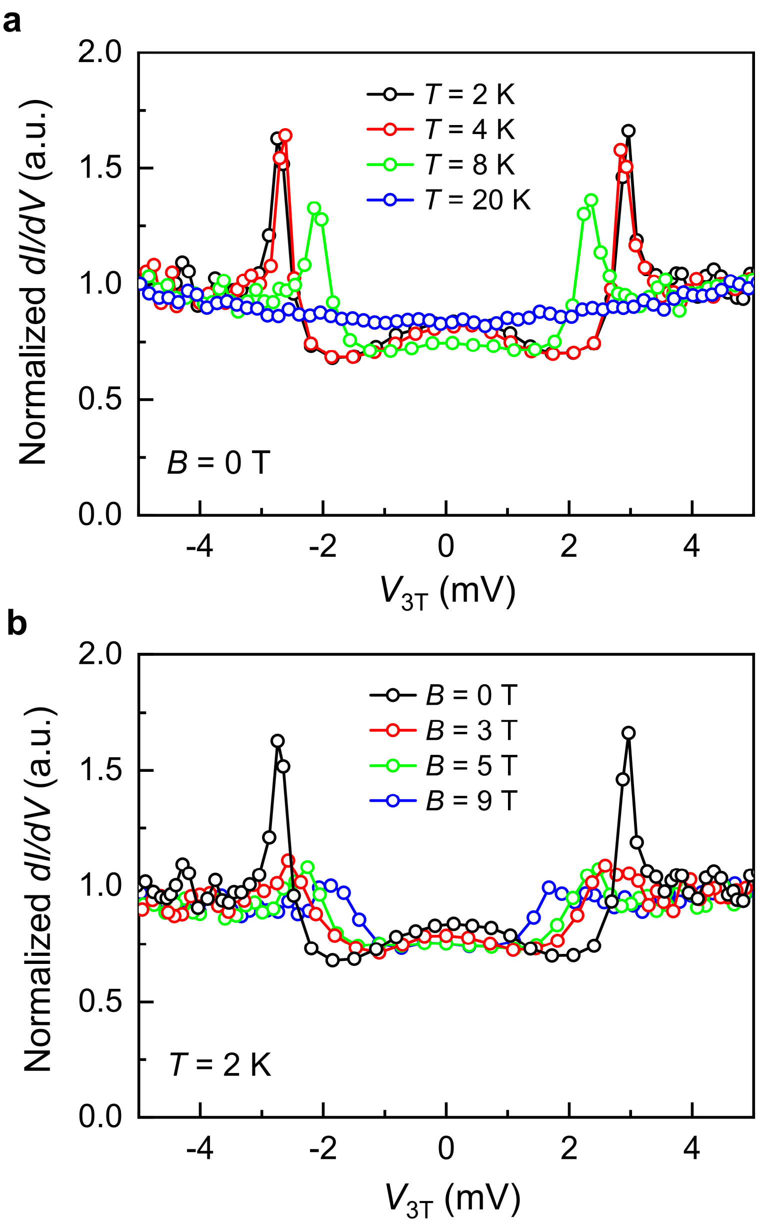

Supplementary Figure 6. Bias dependence of the conductance (*dI/dV*) at the $Fe_{0.29}TaS_2$/SC interface. **a**, The *dI/dV* curves at temperatures from $T = 2$ K to $T = 20$ K at $\mathbf{B} = 0$ T. **b,** The *dI/dV* curves at magnetic fields from $\mathbf{B} = 0$ T to $B = 9$ T at $T = 2$ K. The results were obtained on the device C.

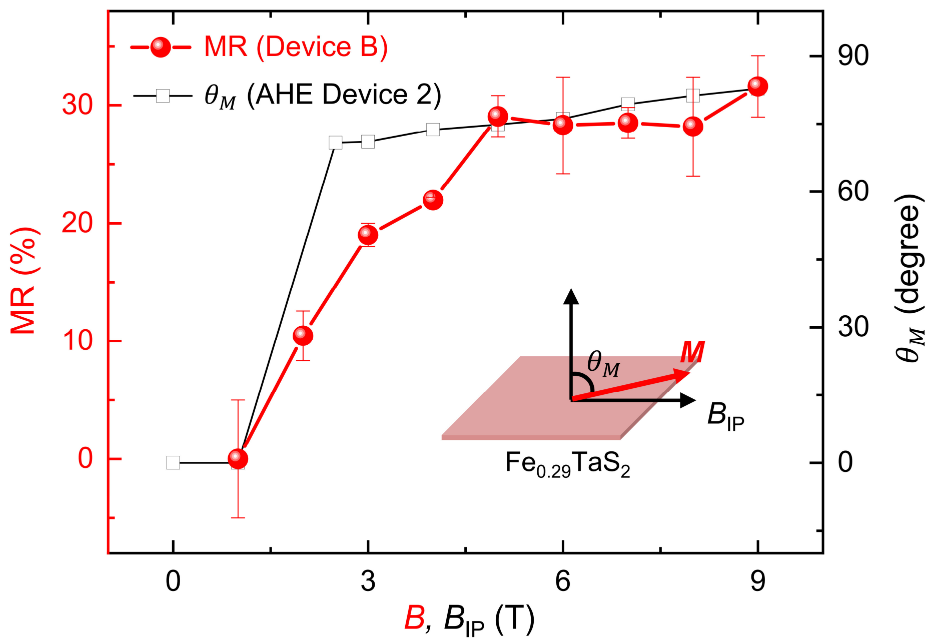

Supplementary Figure 7. The MR of $Fe_{0.29}TaS_2$/NbN as a function of the external magnetic field. The red dots represent the MR measured on the device B at $T = 2$ K and $V_{3T} \sim 0.4$ mV, and the open black squares represent the magnetization angle as a function of the in-plane magnetic field at $T = 2$ K.

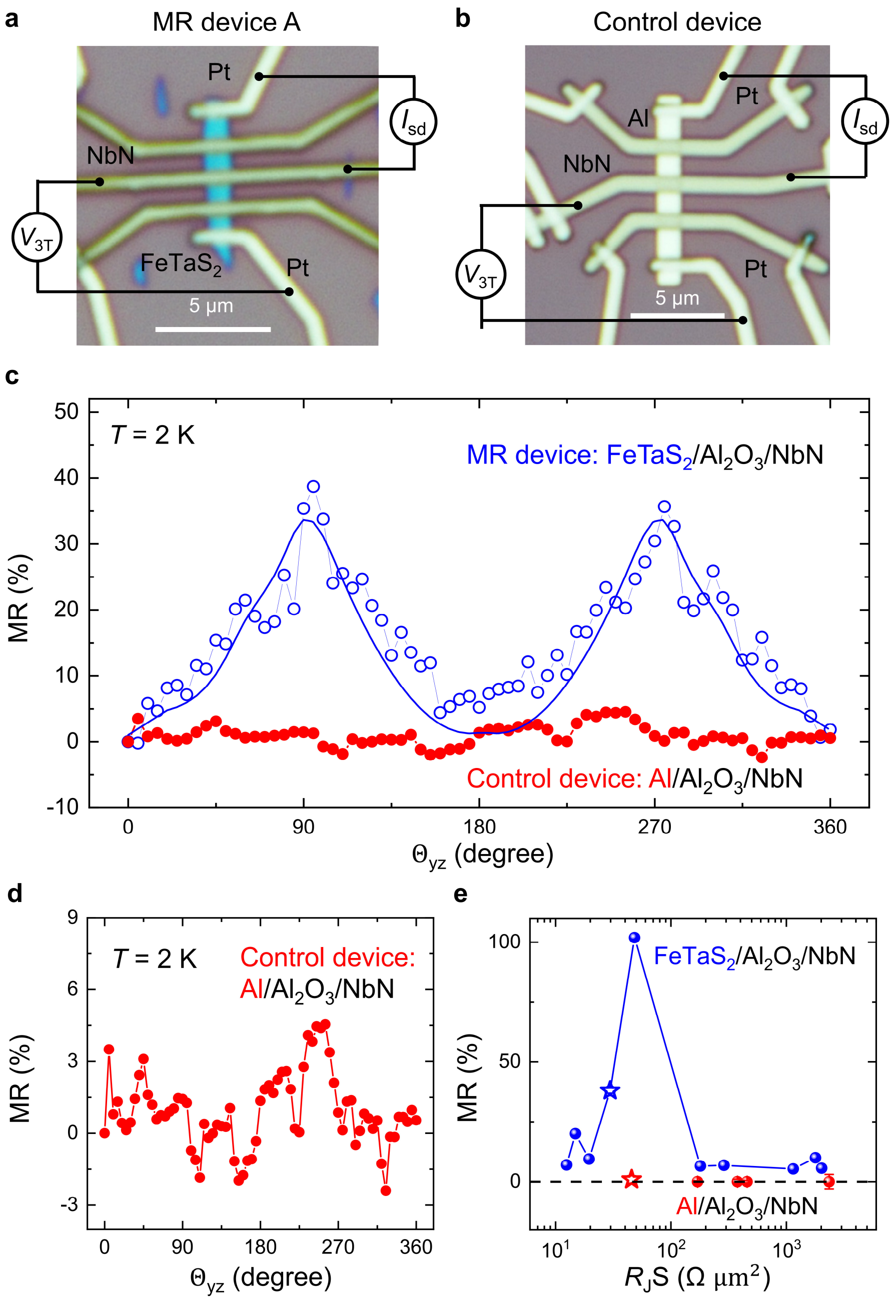

Supplementary Figure 8. Comparison of spin-triplet MR and SC vortex-induced MR. a, b, The optical images of the spin-triplet MR device (device A) and the control device that measures the vortex-induced MR and their measurement geometry. For the control device, everything is the same as the spin-triplet MR device except that the 2D vdW FM $Fe_{0.29}TaS_2$ flake is replaced by a 20 nm Al electrode. **c,** The comparison of the spin-triplet MR (blue symbols) and vortex-induced MR (red symbols) as a function of the magnetic field angle measured at $B = 9$ T and $T = 2$ K with

V_{bias} = 1mV. The interfacial resistance area product ($R_J S$) is 29.7 and 45.6 Ω μm^2 for spin-triplet MR device and control device, respectively. **d,** The MR results of control device (the same curve as in **c**). Clearly, the signal is much smaller and within the noise level. **e,** The comparison of MR in control (red symbols) and spin-triplet MR devices (blue symbols) with various $R_J S$ values. The blue and red stars correspond to the results of spin-triplet MR and control devices in **c**, respectively.

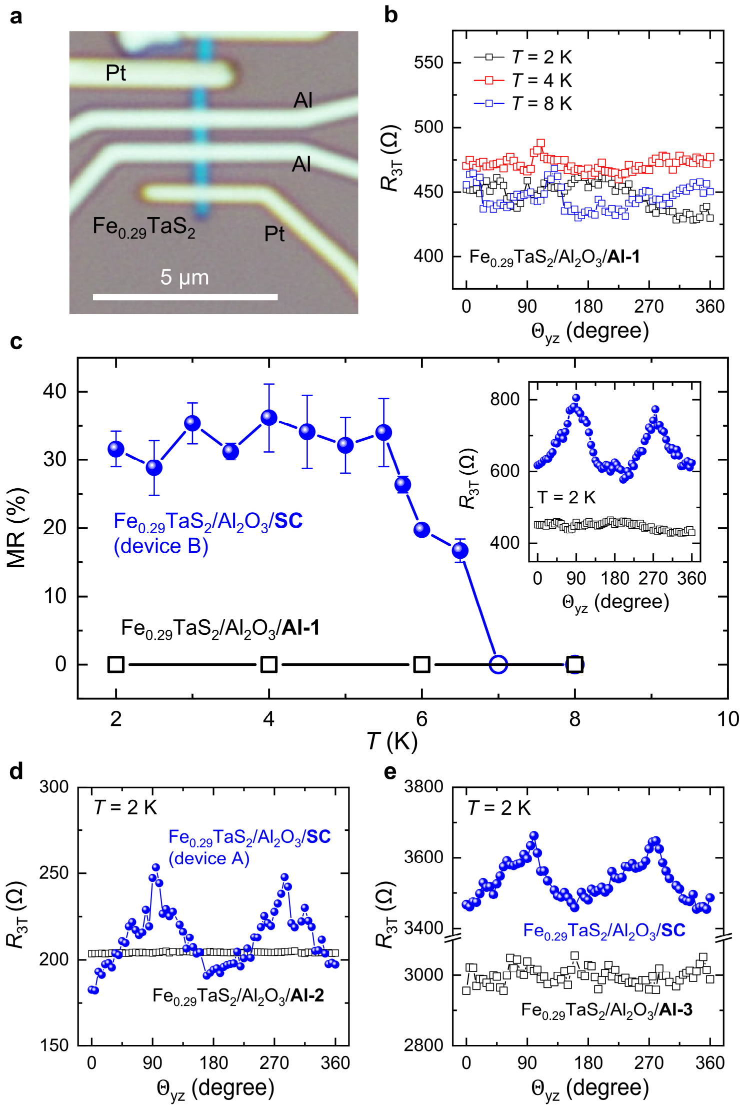

Supplementary Figure 9. Results of control devices of $Fe_{0.29}TaS_2/Al_2O_3$/normal metal (Al). a, The optical images of a typical control device $Fe_{0.29}TaS_2/Al_2O_3/Al$, where the SC NbN electrode is replaced with ~ 50 nm Al. **b,** The MR results of the control device $Fe_{0.29}TaS_2$/ Al_2O_3/Al-1 measured at $B = 9$ T and $T = 2$, 4, and 8 K, respectively. c, The comparison of the spin-triplet MR (blue symbols) and control device $Fe_{0.29}TaS_2/Al_2O_3/Al$ (black symbols; R_JS: 59.4 Ω μm^2) as a function of temperature measured at $B = 9$ T. Inset: The MR curves of control device and the spin-triplet MR device B with similar R_JS. **d, e,** The absence of MR signals on two other control devices $Fe_{0.29}TaS_2/Al_2O_3$/Al-2, and $Fe_{0.29}TaS_2/Al_2O_3$/Al-3 with R_JS of 33.8 and 1626.2 Ω μm^2, respectively, measured at $B = 9$ T and $T = 2$ K. The blue symbols represent the MR curves on spin-triplet MR devices with similar R_JS.

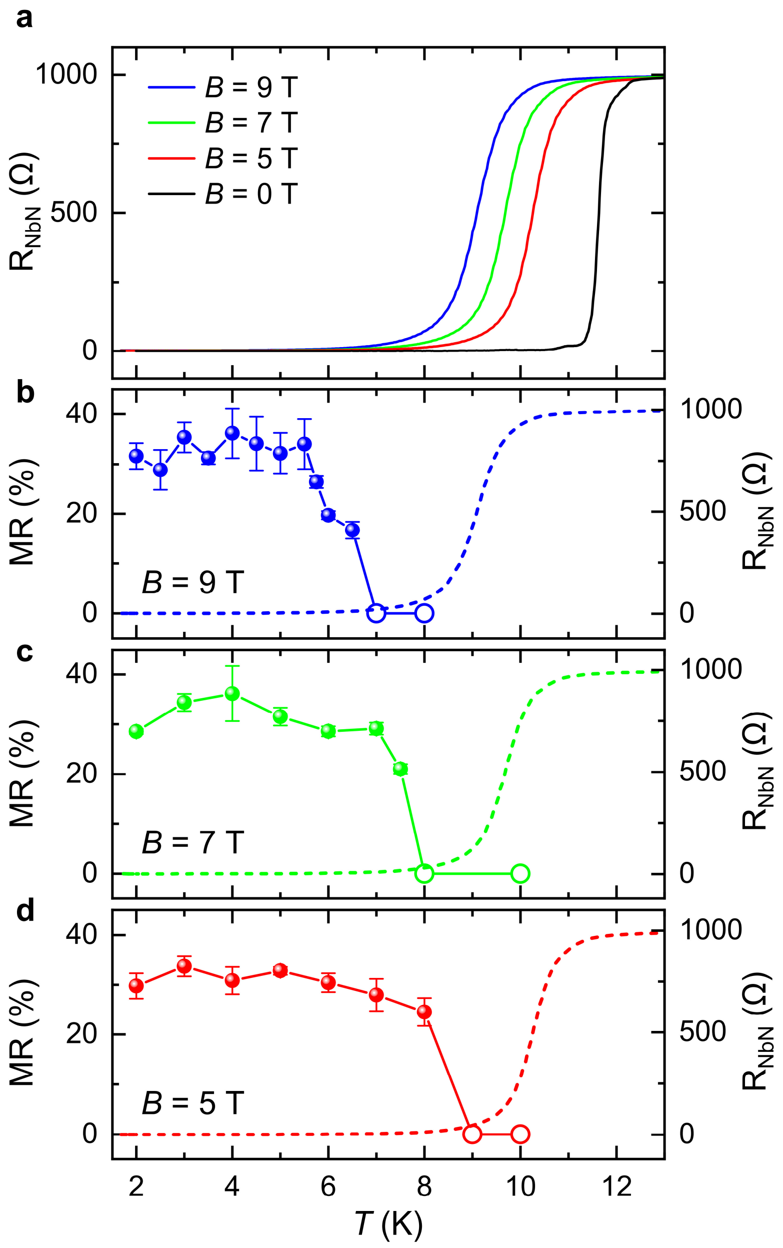

Supplementary Figure 10. The T_{SC} of the NbN electrode and the correlation with the temperature-dependent MR. a, The temperature dependence of the resistance measured on a typical NbN electrode under the perpendicular B = 0, 5, 7, and 9 T, respectively. **b-d,** The temperature dependence of MR and the resistance of the NbN electrode (dashed lines) measured at B = 9, 7, and 5 T, respectively.
